# Acquired cerebral amyloid angiopathy: more questions than answers

**DOI:** 10.1111/ene.16299

**Published:** 2024-04-10

**Authors:** Simon Fandler‐Höfler, Benedetta Storti

**Affiliations:** ^1^ Department of Neurology Medical University of Graz Graz Austria; ^2^ Cerebrovascular Unit Fondazione IRCCS Istituto Neurologico Carlo Besta Milan Italy

Cerebral amyloid angiopathy (CAA) is defined by the deposition of amyloid β (Aβ) in cerebral vessel walls [1]. Until rather recently, two forms of CAA were known: sporadic CAA, commonly found in advanced age; and hereditary CAA, caused by monogenic mutations, most frequently in the Aβ precursor protein.

In the last few years, an acquired form of CAA, often termed iatrogenic CAA, has been described. The underlying aetiological hypothesis centres on the transmission of pathological Aβ from an exogenous source such as contaminated medical products, later disseminating in the central nervous system and causing CAA [2]. Transmission of exogenous amyloid in humans was initially observed in association with iatrogenic Creutzfeldt–Jakob disease [3], and only later also in the absence of it [2]. To date, many individual cases and some case series of iatrogenic CAA have been reported, mostly consisting of patients with childhood neurosurgery and occurrence of symptomatic CAA after a latency period of several decades [4, 5]. The main described sources for transmission of pathological Aβ were human cadaveric‐derived lyophilized dura derivates used as patch and embolization material in neurosurgical procedures, the uses of which had stopped in the late 1980s due to the recognized association of those products with iatrogenic Creutzfeldt–Jakob disease.

Whilst diagnostic criteria for possible and probable iatrogenic CAA have been proposed [6], it is not entirely clear how to differentiate patients with iatrogenic and sporadic CAA. Although the suspicion for iatrogenic CAA is much higher in patients with early onset of symptoms (<55 years) and previous exposure to a potential source of pathological Aβ, particularly lyophilized dura, the differentiation is much more difficult in patients with neurosurgery in adulthood and consecutive occurrence of CAA at greater ages.

In this issue of the *European Journal of Neurology*, Panteleienko et al. report five cases of patients with suspected iatrogenic CAA in adults over the age of 65 [7]. This report underlines the difficulty in the diagnosis of iatrogenic CAA in older individuals, who present at an age compatible with the sporadic form, as suspicion for acquired causes of CAA is not usually raised in this age group. Limitations include that only one of the five patients had confirmed use of cadaveric dura and amyloid deposition in the central nervous system was not confirmed. Whilst it appears more than plausible that not only patients with early onset of CAA‐related symptoms may have iatrogenic CAA, there are no definitive pointers to distinguish iatrogenic CAA in older individuals from sporadic CAA with a coincidental history of potential exposure to pathological Aβ.

Recently, another potential cause of transmissible CAA has drawn attention. In a large retrospective study using nationwide health registry and blood bank data in Sweden and Denmark, associations of the risk of intracerebral haemorrhage between donors and recipients of red blood cell products were investigated [8]. Sparking interest, the authors found that receiving blood from a donor who (later) developed multiple intracerebral haemorrhages was associated with a higher rate of brain haemorrhages in the recipient. Whilst there are some relevant limitations to this study, including missing information on CAA and its related features, one important hypothesis based on this study's results is the potential of a transmission of pathological Aβ from donor to recipient, conveying an increased risk of CAA (and consecutive intracerebral haemorrhage). So far, no cases of individual patients with CAA potentially linked to blood transfusions had been reported.

In this issue of the *European Journal of Neurology*, Kaushik et al. report two cases of patients with CAA and a history of blood transfusion as infants [9]. Both presented with CAA at a reasonably young age (47 and 57 years), had severe findings of CAA in neuroimaging and no genetic causes of Aβ disease. These two patients were identified in a CAA outpatient clinic, where all patients had recently been asked about previous blood transfusions (as a reaction to the previously discussed study), with blood transfusions reported in extended medical history in those two patients (6% of those in whom it was assessed). Again, there are important limitations: Amyloid deposition was not confirmed through cerebrospinal fluid analysis or positron emission tomography, the CAA status of blood donors could not be assessed, and the coincidence of blood transfusion alone and CAA in later life does not prove any causality. Whilst potential for pathological Aβ transmission through peripheral injections has been shown in mice [10], much more evidence is needed to confirm a link between blood transfusions and the occurrence of CAA. The authors themselves discuss this and conclude that the most likely diagnosis in these two cases may be early‐onset sporadic (rather than acquired) CAA.

In summary, these two reports underline the difficulty of diagnosis of acquired CAA. Although there is solid pathophysiological evidence of amyloid transmissibility, it is unclear how to fully differentiate acquired from other forms of CAA, and it is further unclear whether such a differentiation carries any direct clinical implication apart from increasing the understanding of transmission pathways, potentially leading to actions to counteract them. Larger quantitative studies are needed to assess the clinical course of acquired CAA in comparison to sporadic CAA, but also to investigate potential differences in biomarkers, neuroimaging or clinical factors, which could give important insights in the diagnosis and prognosis of acquired CAA.

## AUTHOR CONTRIBUTIONS

**Simon Fandler‐Höfler:** Writing – original draft. **Benedetta Storti:** Writing – review and editing.

## CONFLICT OF INTEREST STATEMENT

The authors report no competing interests in connection with this article.

## Data Availability

Data sharing not applicable to this article as no datasets were generated or analysed during the current study.
